# Assessment of Shared Decision-making for Stroke Prevention in Patients With Atrial Fibrillation: A Randomized Clinical Trial

**DOI:** 10.1001/jamainternmed.2020.2908

**Published:** 2020-07-20

**Authors:** Marleen Kunneman, Megan E. Branda, Ian G. Hargraves, Angela L. Sivly, Alexander T. Lee, Haeshik Gorr, Bruce Burnett, Takeki Suzuki, Elizabeth A. Jackson, Erik Hess, Mark Linzer, Sarah R. Brand-McCarthy, Juan P. Brito, Peter A. Noseworthy, Victor M. Montori

**Affiliations:** 1 Knowledge and Evaluation Research Unit, Mayo Clinic, Rochester, Minnesota; 2 Department of Biomedical Data Sciences, Leiden University Medical Center, Leiden, the Netherlands; 3 Department of Biostatistics and Informatics, Colorado School of Public Health, Anschutz Medical Campus, University of Colorado Denver, Aurora; 4 Division of Biomedical Statistics and Informatics, Department of Health Sciences Research, Mayo Clinic, Rochester, Minnesota; 5 Division of General Internal Medicine, Hennepin Health, Minneapolis, Minnesota; 6 Thrombosis Clinic and Anticoagulation Services, Park Nicollet Health Services, St Louis Park, Minnesota; 7 Division of Cardiology, Department of Medicine, University of Mississippi Medical Center, Jackson; 8 Department of Internal Medicine, Division of Cardiovascular Disease, University of Alabama at Birmingham, Birmingham; 9 Department of Emergency Medicine, University of Alabama at Birmingham, Birmingham; 10 Department of Psychiatry and Psychology, Mayo Clinic, Rochester, Minnesota; 11 Robert D. and Patricia E. Kern Center for the Science of Health Care Delivery, Mayo Clinic, Rochester, Minnesota; 12 Heart Rhythm Services, Department of Cardiovascular Diseases, Mayo Clinic, Rochester, Minnesota

## Abstract

**Importance:**

Shared decision-making (SDM) about anticoagulant treatment in patients with atrial fibrillation (AF) is widely recommended but its effectiveness is unclear.

**Objective:**

To assess the extent to which the use of an SDM tool affects the quality of SDM and anticoagulant treatment decisions in at-risk patients with AF.

**Design, Setting, and Participants:**

This encounter-randomized trial recruited patients with nonvalvular AF who were considering starting or reviewing anticoagulant treatment and their clinicians at academic, community, and safety-net medical centers between January 30, 2017 and June 27, 2019. Encounters were randomized to either the standard care arm or care that included the use of an SDM tool (intervention arm). Data were analyzed from August 1 to November 30, 2019.

**Interventions:**

Standard care or care using the Anticoagulation Choice Shared Decision Making tool (which presents individualized risk estimates and compares anticoagulant treatment options across issues of importance to patients) during the clinical encounter.

**Main Outcomes and Measures:**

Quality of SDM (which included quality of communication, patient knowledge about AF and anticoagulant treatment, accuracy of patient estimates of their own stroke risk [within 30% of their estimate], decisional conflict, and satisfaction), decisions made during the encounter, duration of the encounter, and clinician involvement of patients in the SDM process.

**Results:**

The clinical trial enrolled 922 patients (559 men [60.6%]; mean [SD] age, 71 [11] years) and 244 clinicians. A total of 463 patients were randomized to the intervention arm and 459 patients to the standard care arm. Participants in both arms reported high communication quality, high knowledge, and low decisional conflict, demonstrated low accuracy in their risk perception, and would similarly recommend the approach used in their encounter. Clinicians were significantly more satisfied after intervention encounters (400 of 453 encounters [88.3%] vs 277 of 448 encounters [61.8%]; adjusted relative risk, 1.49; 95% CI, 1.42-1.53). A total of 747 of 873 patients (85.6%) chose to start or continue receiving an anticoagulant medication. Patient involvement in decision-making (as assessed through video recordings of the encounters using the Observing Patient Involvement in Decision Making 12-item scale) scores were significantly higher in the intervention arm (mean [SD] score, 33.0 [10.8] points vs 29.1 [13.1] points, respectively; adjusted mean difference, 4.2 points; 95% CI, 2.8-5.6 points). No significant between-arm difference was found in encounter duration (mean [SD] duration, 32 [16] minutes in the intervention arm vs 31 [17] minutes in the standard care arm; adjusted mean between-arm difference, 1.1; 95% CI, −0.3 to 2.5 minutes).

**Conclusion and Relevance:**

The use of an SDM encounter tool improved several measures of SDM quality and clinician satisfaction, with no significant effect on treatment decisions or encounter duration. These results help to calibrate expectations about the value of implementing SDM tools in the care of patients with AF.

**Trial Registration:**

ClinicalTrials.gov Identifier: NCT02905032

## Key Points

**Question:**

Does use of the Anticoagulation Choice Shared Decision Making encounter tool affect the quality of shared decision-making and anticoagulant treatment selection in patients with atrial fibrillation who are at risk of experiencing stroke?

**Findings:**

In this randomized clinical trial of 922 patients with atrial fibrillation and 151 clinicians, use of the Anticoagulation Choice Shared Decision Making encounter tool resulted in several improvements in markers of shared decision-making quality and clinician satisfaction without changing anticoagulant treatment rates or encounter length.

**Meaning:**

The results indicate that use of a tool for shared decision-making in the clinical encounter contributes to the care of patients with atrial fibrillation who are considering anticoagulant treatment.

## Teaser

This randomized clinical trial assesses the use of a shared decision-making tool during clinical encounters and the effects on anticoagulant treatment decisions in patients with atrial fibrillation who are at risk of experiencing stroke.

## Introduction

Atrial fibrillation (AF) is the most common cardiac arrhythmia observed in clinical practice, with more than 5 million people experiencing AF in the US alone.^1,2^ Atrial fibrillation is associated with increased stroke and systemic embolism rates and increased morbidity and mortality.^1^ Anticoagulant treatment reduces the risk of stroke by approximately 65% in patients with nonvalvular AF.^3^ Almost one-half of patients at risk of experiencing stroke do not start, and a similar proportion do not continue, to receive anticoagulant treatment and experience preventable strokes.^4,5,6,7^ This gap in care is likely multifactorial. It may reflect misunderstandings about the risk of stroke or the association of that risk with anticoagulant treatment (warfarin or direct oral anticoagulant [DOAC] medications), or it may result from concerns about bleeding, activity, diet and drug interactions, anticoagulant treatment reversal, out-of-pocket costs, or the need for periodic monitoring. Some patients may not be able to use anticoagulant medications safely and consistently.

In 2014, 3 major cardiovascular organizations formulated guidelines and issued a class 1 recommendation for the use of shared decision-making (SDM) to individualize the anticoagulant treatment of patients with nonvalvular AF who are at risk of experiencing stroke.^8^ To implement this recommendation, several tools to facilitate SDM among patients with AF have been developed.^9,10,11,12,13^ However, most of these tools have not been rigorously evaluated, omit DOAC medications, present outdated data, do not directly support the patient-clinician conversation, or do not address practical considerations that are important to the success of ongoing safe anticoagulant treatment, such as leisure activities, diet, travel, and out-of-pocket costs.^9,11,14,15,16^

To address these limitations and support at-risk patients with AF and their clinicians in making decisions about anticoagulant treatment, we developed the Anticoagulation Choice Shared Decision Making tool.^17,18^ The aim of the current study was to assess the extent to which the use of the Anticoagulation Choice Shared Decision Making tool affects the quality of SDM and anticoagulant treatment decisions in patients with AF who are at risk of experiencing stroke.

## Methods

### Design, Setting, and Participants

This encounter-level multicenter randomized clinical trial compared the use of standard care during the clinical encounter with the use of the Anticoagulation Choice Shared Decision Making tool (which presents individualized risk estimates and compares anticoagulant treatment options across issues of importance to patients) during the clinical encounter to examine the effects of the 2 approaches on SDM and clinical outcomes. This report addresses the outcome data collected during and immediately following the index clinical encounter. The institutional review boards at the coordinating center (Mayo Clinic) and other participating sites (Hennepin Health, Park Nicollet Health Partners, the University of Alabama at Birmingham, and the University of Mississippi Medical Center) approved the study procedures, and the study protocol for the clinical trial was published previously.^17^ The trial protocol is available in Supplement 1. Written informed consent was obtained from all participants. The study followed the Consolidated Standards of Reporting Trials (CONSORT) reporting guideline for randomized clinical trials.

The clinical trial took place in emergency and inpatient hospital departments and outpatient safety-net, primary care, and cardiology clinics at US academic medical centers. Participant recruitment began at an academic medical center (Mayo Clinic), a suburban group practice (Park Nicollet Health Partners), and an urban safety-net health system (Hennepin Health) in Minnesota in January 2017. The University of Alabama at Birmingham and the University of Mississippi Medical Center joined the study in December 2018.

All clinicians at the participating sites who regularly had conversations about anticoagulant treatment with patients with AF were eligible for participation. Participating clinicians provided written informed consent before enrolling patients. Adult patients (aged ≥18 years) were eligible for participation if they were able to read and understand the informed consent document, had a diagnosis of nonvalvular AF, and were at high risk of experiencing a thromboembolic event. The risk of a thromboembolic event was measured using the CHA_2_DS_2_-VASc score (congestive heart failure, hypertension, age ≥75 years, diabetes, previous stroke or transient ischemic attack or thromboembolism, vascular disease, age 65-74 years, and sex category; score range, 0-9, with higher scores indicating higher risk); a CHA_2_DS_2_-VASc score of 1 or more for men and 2 or more for women indicated high risk.

Patients were classified into 1 of 2 cohorts: start and review. The start cohort comprised patients who were new to the receipt of anticoagulant treatment (ie, had not received anticoagulant treatment within the past 6 months). During the clinical trial, the practice of prescribing a DOAC medication in the emergency department and referring the patient to an outpatient clinic for full discussion of anticoagulant treatment led us to modify the protocol by including in the start cohort all patients who had been prescribed an anticoagulant medication within 10 days of the clinical trial encounter but were otherwise new to the receipt of anticoagulant treatment. The review cohort comprised patients who were currently receiving ongoing anticoagulant medication or who had received anticoagulant medication within the past 6 months.

### Randomization and Intervention

Encounters were randomized on a 1:1 ratio to either standard care or care that included use of the SDM tool, which allowed clinicians to participate in both study arms. The randomization algorithm (generated within the Remote Data Capture [REDCap] software system; Vanderbilt University), which was built by the clinical trial statistician (M.E.B.), used a stratified block randomization with blocks of random size. The clinical trial was stratified by medical center, cohort (start vs review), and stroke risk (CHA_2_DS_2_-VASc score of 1 for men and 2 for women vs >1 for men and >2 for women).

In the standard care arm, clinical encounters were conducted according to the clinicians’ usual approach. In the intervention arm, clinicians were asked to use the Anticoagulation Choice Shared Decision Making tool in their encounters. This tool is a freely available online conversation aid that is designed for use within the encounter.^19,20^ The tool calculates the patient’s risk of stroke using the CHA_2_DS_2_-VASc score^21^ and provides the patient’s individualized risk of experiencing stroke at 1 year or 5 years, with and without anticoagulant treatment, using natural frequency expressions (eg, “out of 100 people like you”) and 100-person pictographs that illustrate the proportion of people experiencing nondisabling strokes, disabling or fatal strokes, or no such events. The tool then supports the comparison of available anticoagulant treatment options (ie, warfarin and DOAC medications) across patient-important issues, such as how to use the medications, the need for periodic monitoring, the reversibility of anticoagulant treatment, the estimated out-of-pocket costs, and the association of lifestyle or medical factors with the risk of bleeding (using the HAS-BLED [hypertension, abnormal kidney or liver function, stroke, bleeding, labile international normalized ratio, elderly age (>65 years), and drug or alcohol use] estimator; score range, 0-9, with higher scores indicating higher risk^22^). The tool offers a patient report and tailored text that can be copied into the clinical note to document the conversation and the decision. Participating clinicians at each site completed a training session with a study coordinator, including an overview of the Anticoagulation Choice Shared Decision Making tool and a video tutorial about its intended use.

### Outcomes

Clinicians completed a baseline survey at enrollment, and both patients and clinicians completed a survey immediately after the clinical encounter (eMethods in Supplement 2). The survey captured patients’ sociodemographic characteristics, health literacy (measured by a series of screening questions, with inadequate health literacy defined as a patient self-report of being “not at all” or “a little bit” confident in filling out medical forms without assistance),^23^ and subjective numeracy (measured by the Subjective Numeracy Scale; score range, 1-6, with higher scores indicating higher subjective numeracy).^24^ With the participant’s written consent, the encounter was recorded (either audiovisual or audio only).

#### Participant-Reported Outcomes

The primary outcome was the quality of SDM, a multidimensional concept that requires high-quality communication, effective knowledge transfer to the patient, agreement between the patient and the clinician on the course of action selected at the end of the encounter, and satisfaction with the decision-making process. The Consumer Assessment of Healthcare Providers and Systems Clinician and Group Survey was used to assess the quality of communication.^25^ Each item was coded as yes (definitely or somewhat) or no. Six questions about AF and anticoagulant treatment were used to assess knowledge transfer. To assess the accuracy of patients’ estimations of their own stroke risk, we asked patients to provide the number of people like them (out of 100 people) who they perceived could be expected to have a stroke within the next year. We considered a correct response any answer that was within either 10% (strict threshold) or 30% (liberal threshold) of the respondent’s actual CHA_2_DS_2_-VASc risk score. Comparison of the patient’s and clinician’s reported course of action was used to assess decision concordance.

Decisional satisfaction was assessed using the Decisional Conflict Scale (score range, 0-100, with higher scores indicating greater decisional conflict), which reflected the degree of uncertainty about the choice.^26^ Participants indicated, on a 7-point Likert scale (with higher scores indicating stronger recommendation), the extent to which they would recommend the approach used in the encounter to other patients and clinicians. Clinicians indicated, on a 5-point Likert scale (with higher scores indicating greater satisfaction), the extent to which they were satisfied with their conversation with the patient. Each question was converted to a binary response of strongly recommend (6-7 points) or completely satisfied (4-5 points), respectively.

#### Observed Encounter Outcomes

After training and documentation of reliability, reviewers from the study team, working independently and in duplicate, used the Observing Patient Involvement in Decision Making 12-item (OPTION12) scale (score range, 0-100, with 0 indicating minimal behavior and 100 indicating maximal behavior) to code clinicians’ behavior to involve patients in decision-making.^27^ Interrater reliability (using the Lin concordance correlation coefficient [CCC]) was verified at baseline, at 33% of encounter recordings, and at 66% of encounter recordings (Lin CCC range, 0.84%-0.96%).

Similarly, reviewers coded user fidelity (ie, use of the conversation aid as intended) using an ad hoc scale with adequate reliability (ie, κ interrater agreement of 0.8-1.0 across items). On-site study coordinators captured the duration (measured in minutes) of the full encounter. When unavailable, the duration documented in the recording of the encounter was used.

### Statistical Analysis

The study was conducted and analyzed according to the intention-to-treat principle, which included all encounters in the arm to which they were randomly assigned. The primary analysis was conducted at the encounter level using mixed-effects models that were adjusted by arm, cohort (start vs review), and stroke risk (CHA_2_DS_2_-VASc score of 1 for men and 2 for women vs >1 for men and >2 for women), with the random effect of clinic and clinician.^28^ The 15 participants who correctly answered 3 or fewer knowledge questions were grouped into 1 category, with the remaining participants grouped into 3 categories based on the exact number of correct answers (4, 5, or 6 answers). Knowledge was modeled as correct responses (successes) out of 6 questions as a mixed-effects logistic regression analysis with a family of binomial distribution, adjusted by arm, cohort, and stroke risk. Assumptions for all models were verified, with no deviations found.

The clinical trial recruitment goal was 1000 patient encounters (500 encounters per arm) based on previously reported sample size estimations.^17^ As conducted, the clinical trial produced estimates of between-arm differences with a margin of error (one-half of the 95% CI) of 1.9 (out of 100) for the Decisional Conflict Scale, 1.4 (out of 100) for the OPTION12 scale, and 1.4 minutes for the encounter duration. For binary outcomes, the margin of error ranged from 3% to 9%, with patient knowledge estimates having a margin of error of 14%.

Missing outcome data owing to the nonreturn of surveys or incomplete survey responses occurred in 2% to 7% of encounters across outcomes. According to the statistical plan, outcomes associated with encounter recordings were not imputed because we considered it inappropriate to assume patients or clinicians chose not to be recorded at random. Multiple imputation was conducted with data that were treated as missing at random,^29^ data with 5 imputations, data with relative efficiency ranging from 98% to 99%, and data within range values.

The exploration of heterogeneity of treatment effect included all SDM outcomes using the χ^2^ test statistic of the differences in the log likelihood to test interactions by type of clinic (academic, community, or safety net), by cohort (start or review), by stroke risk (CHA_2_DS_2_-VASc score of >1 for men or >2 for women), and by numeracy (mean score on the Subjective Numeracy Scale of ≤4 points or >4 points, with >4 points considered adequate numeracy). The Benjamini-Hochberg method was used to account for multiple comparisons.^30^ All tests were 2-sided and unpaired, and data analysis was conducted using Stata statistical software, version 15 (StataCorp LLC). Data were analyzed from August 1 to November 30, 2019.

A data and safety monitoring board met before study initiation to approve the board’s charter and met biannually thereafter. The board monitored study conduct, data quality, and safety signals, although no interim efficacy analyses were planned or conducted. On review of the results, the board released the data for publication.

## Results

### Participant Characteristics

Study recruitment occurred from January 30, 2017, to June 27, 2019. Patients and clinicians consented to participate in 942 of the 1827 eligible encounters (52%). A total of 922 patients and 244 clinicians were enrolled and included in the analyses. Among patients, 463 individuals (291 men [62.9%]; mean [SD] age, 71 [11] years) were randomized to the intervention arm, and 459 individuals (268 men [58.4%]; mean [SD] age, 71 [10] years) were randomized to the standard care arm. Among clinicians, 112 of 222 individuals (50.5%) were women, with a mean (SD) age of 43 (12) years (Table 1, Table 2, and Figure).

**Table 1.** Patient Characteristics

Characteristic	No./total No. (%)	
	Intervention arm	Standard care arm
Total, No.	463	459
Age, mean (SD)	71 (11)	71 (10)
Sex		
Male	291/463 (62.9)	268/459 (58.4)
Female	172/463 (37.1)	191/459 (41.6)
Race		
White	387/456 (84.9)	380/450 (84.4)
Black	48/456 (10.5)	54/450 (12.0)
Asian	5/456 (1.1)	5/450 (1.1)
American Indian or Alaskan native	4/456 (0.9)	1/450 (0.2)
Multiple races	10/456 (2.2)	8/450 (1.8)
Other	2/456 (0.4)	2/450 (0.4)
Hispanic	4/452 (0.9)	3/441 (0.7)
Inadequate health literacy^a^	43/448 (9.6)	30/435 (6.9)
SNS Preference subscale score, mean (SD)^b^	4 (1)	4 (1)
SNS Inadequate numeracy score^c^	140/444 (31.5)	136/432 (31.5)
CHA_2_DS_2_-VASc score		
1	35/463 (7.6)	37/459 (8.1)
2	101/463 (21.8)	95/459 (20.7)
3	120/463 (25.9)	112/459 (24.4)
4	96/463 (20.7)	109/459 (23.7)
5	63/463 (13.6)	69/459 (15.0)
6	32/463 (6.9)	22/459 (4.8)
7	11/463 (2.4)	13/459 (2.8)
8	4/463 (0.9)	1/459 (0.2)
9	1/463 (0.2)	1/459 (0.2)
HAS-BLED score		
0	18/463 (3.9)	17/459 (3.7)
1	128/463 (27.6)	114/459 (24.8)
2	180/463 (38.9)	179/459 (39.0)
3	94/463 (20.3)	105/459 (22.9)
4	33/463 (7.1)	37/459 (8.1)
5	10/463 (2.2)	7/459 (1.5)
Cohort		
Start^d^	98/463 (21.2)	99/459 (21.6)
Review	365/463 (78.8)	360/459 (78.4)

Abbreviations: CHA_2_DS_2_-VASc, congestive heart failure, hypertension, age ≥75 years, diabetes, previous stroke or transient ischemic attack or thromboembolism, vascular disease, age 65-74 years, and sex category; HAS-BLED, hypertension, abnormal kidney or liver function, stroke, bleeding, labile international normalized ratio, elderly age (>65 years), and previous drug or alcohol use or medication use predisposing to bleeding; SNS, Subjective Numeracy Scale.

^a^ Inadequate health literacy was defined as a patient self-report of being “not at all” or “a little bit” confident in filling out medical forms without assistance.^31^

^b^ Data were missing for 15 participants in the intervention arm and 24 participants in the standard care arm.

^c^ Inadequate numeracy was defined as a mean SNS score of less than 4 points.

^d^ Patients in the start cohort were treatment naive.

**Table 2.** Clinician Characteristics

Characteristic	No./total No. (%)	
	Participated in study	Had encounter with ≥1 patient enrolled in study
Total, No.	244	151
Age, mean (SD)	43 (12)	45 (13)
Sex		
Male	110/222 (49.5)	75/141 (53.2)
Female	112/222 (50.5)	66/141 (46.8)
Clinician type		
Physician	171/222 (77.0)	111/141 (78.7)
Nurse practitioner	31/222 (14.0)	18/141 (12.8)
Physician assistant	8/222 (3.6)	4/141 (2.8)
Pharmacist	8/222 (3.6)	4/141 (2.8)
Practice type		
Cardiology	45/222 (20.3)	34/141 (24.1)
Cardiac electrophysiology	33/222 (14.9)	27/141 (19.1)
Internal medicine	73/222 (32.9)	35/141 (24.8)
Family medicine	41/222 (18.5)	24/141 (17.0)
Pharmacy	4/222 (1.8)	1/141 (0.7)
In residency or fellowship	59/222 (26.6)	38/141 (27.0)
Clinicians per site, median (range)	54 (5-99)	27 (4-69)
Enrolled patients per clinician, median (range)	1 (0-74)	2 (1-74)

**Figure.**
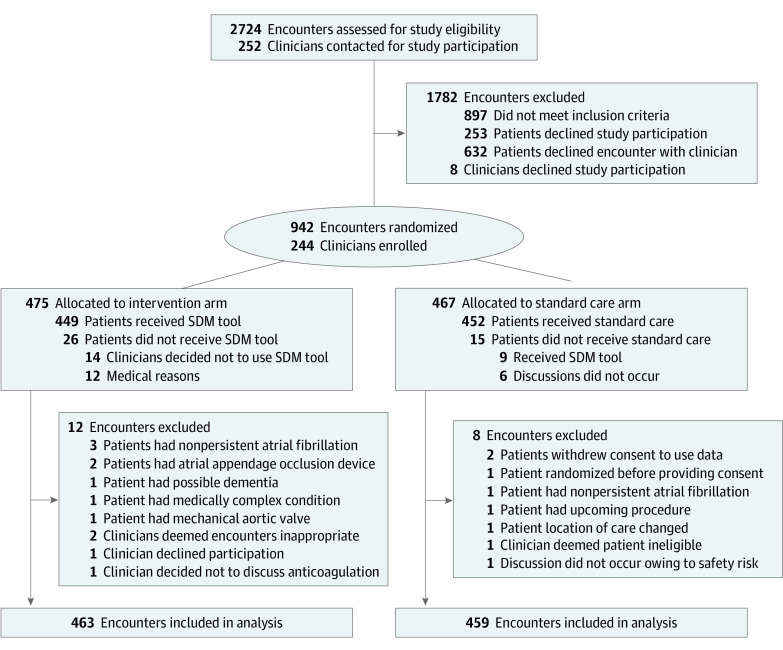
CONSORT Diagram SDM indicates shared decision-making.

All patient factors were balanced across arms. Because no significant interactions were found across any of the planned subgroup analyses (eTable 1 in Supplement 2), including no differential effects by cohort (start vs review) or clinic, the clinical trial results are presented for the whole cohort.

### Participant-Reported Outcomes

Almost all patients in both arms reported that the clinician showed respect (426 of 428 patients [99.5%] in the intervention arm and 427 of 427 patients [100%] in the standard care arm), listened carefully (428 of 430 patients [99.5%] in the intervention arm and 427 of 427 patients [100%] in the standard care arm), and used terms that were easy to understand (431 of 432 patients [99.8%] in the intervention arm and 422 of 425 patients [99.3%] in the standard care arm) during the encounter. Patients in both arms (345 of 445 patients [77.5%] in the intervention arm and 315 of 433 patients [72.7%] in the standard care arm; *P* = .15) correctly answered most questions (5 or 6 correct responses of 6 total questions) about anticoagulant treatment for AF (Table 3).

**Table 3.** Participant-Reported Quality of Shared Decision-making

Outcome	No./total No. (%)		Effect (95% CI)	Intracluster correlation	
	Intervention arm (n = 463)	Standard care arm (n = 459)		Clinic	Clinician/clinic
Quality of communication					
Easy to understand	431/432 (99.8)	422/425 (99.3)	NA	NA	NA
Listens carefully	428/430 (99.5)	427/427 (100)	NA	NA	NA
Shows respect	426/428 (99.5)	427/427 (100)	NA	NA	NA
Knowledge transfer score^a^					
≤3	24/445 (5.4)	30/433 (6.9)	1.01 (1.0 to 1.02)^b^	0	0.003
4	76/445 (17.1)	88/433 (20.3)			
5	207/445 (46.5)	191/433 (44.1)			
6	138/445 (31.0)	124/433 (28.6)			
Knowledge of risk^c^					
Strict threshold	30/445 (6.7)	22/434 (5.1)	1.4 (0.8 to 2.2)^b^	0	0.06
Liberal threshold	49/445 (11.0)	40/434 (9.2)	1.3 (0.8 to 1.8)^b^	0.05	0.10
Patient-clinician decision concordance^d^					
Overall	381/465 (81.9)	369/461 (80.0)	1.0 (0.9 to 1.1)^b^	0.13	0.15
Start or continue warfarin	149/382 (39.0)	139/366 (38.0)	NA	NA	NA
Start or continue DOAC	196/384 (51.0)	190/373 (50.9)	NA	NA	NA
Do not receive anticoagulant medication	7/350 (2.0)	9/450 (2.0)	NA	NA	NA
Start or continue aspirin	1/500 (0.2)	6/300 (2.0)	NA	NA	NA
Delay decision	28/400 (7.0)	24/343 (7.0)	NA	NA	NA
Other	0	1/333 (0.3)	NA	NA	NA
Patient-clinician decision discordance^d^	54/450 (12.0)	56/431 (13.0)	NA	NA	NA
Decisional Conflict Scale score, unadjusted mean (SD)^e^					
Overall	16.6 (14.4)	17.9 (14.9)	−1.2 (−3.2 to 0.6)^f^	0.06	0.07
Informed subscale	18.0 (16.2)	20.7 (17.8)	−2.7 (−6.1 to 0.7)^f^	NA	NA
Values subscale	16.6 (16.1)	18.8 (17.1)	−2.2 (−5.2 to 0.9)^f^	NA	NA
Support subscale	14.2 (14.9)	14.3 (14.7)	−0.3 (−2.2 to 1.6)^f^	NA	NA
Uncertainty subscale	18.6 (18.6)	19.6 (19.0)	−1.1 (−3.5 to 1.4)^f^	NA	NA
Effective subscale	15.9 (16.0)	16.3 (16.2)	−0.7 (−2.8 to 1.4)^f^	NA	NA
Patient recommends information-sharing approach to others	390/429 (90.9)	378/425 (88.9)	1.0 (0.97 to 1.1)^b^	0.14	0.24
Clinician recommends information-sharing approach to others	396/453 (87.4)	199/448 (44.4)	2.1 (2.0 to 2.2)^b^	0.22	0.52
Clinician satisfied with discussion	400/453 (88.3)	277/448 (61.8)	1.49 (1.42 to 1.53)^b^	0.16	0.43

Abbreviations: CHA_2_DS_2_-VASc, congestive heart failure, hypertension, age ≥75 years, diabetes, previous stroke or transient ischemic attack or thromboembolism, vascular disease, age 65-74 years, and sex category; DOAC, direct oral anticoagulant; NA, not applicable.

^a^ Six questions about atrial fibrillation and anticoagulant treatment were used to assess knowledge transfer (score range, 0 to 6, with higher scores indicating greater knowledge).

^b^ Adjusted relative risk. Adjusted by treatment arm, cohort (start vs review), and stroke risk (CHA_2_DS_2_-VASc score of 1 vs ≥2 for men and 1-2 vs ≥3 for women), with the random effect of clinic and clinician.

^c^ Patients were asked to provide the number of people like them (of 100 people) whom they expected to experience a stroke within the next year. Patients’ estimates were compared with their actual CHA_2_DS_2_-VASc risk score. A correct response was considered any answer that was within either 10% (strict threshold) or 30% (liberal threshold) of the respondent’s actual CHA_2_DS_2_-VASc risk score.

^d^ Data were missing for 28 participants in the intervention arm and 34 patients in the standard care arm. Clinician and patient responses were paired; therefore, the total numbers in this category varied, as not all patients who were missing a response aligned with clinicians who were missing a response.

^e^ The Decisional Conflict Scale was used to measure decisional satisfaction. Data were missing for 31 participants in the intervention arm and 31 patients in the standard care arm.

^f^ Adjusted mean difference between the intervention and standard care arms. Adjusted by study arm, cohort (start vs review), and stroke risk (CHA_2_DS_2_-VASc score of 1 vs ≥2 for men and 1-2 vs ≥3 for women), with the random effect of clinic and clinician.

No significant difference was observed between study arms with regard to patients’ accuracy of their own perceived risk of stroke using both the strict threshold (30 of 445 patients [6.7%] in the intervention arm vs 24 of 434 patients [5.1%] in the standard care arm; adjusted relative risk [aRR], 1.4; 95% CI, 0.8-2.2) and the liberal threshold (49 of 445 patients [11.0%] in the intervention arm vs 40 of 434 patients [9.2%] in the standard care arm; aRR, 1.3; 95% CI, 0.8-1.8). Decisional conflict was low (Decisional Conflict Scale unadjusted mean [SD] score, 16.6 [14.4] points in the intervention arm and 17.9 [14.9] points in the standard care arm), and patient-clinician concordance about treatment selection was high in both arms (381 of 434 patients [87.8%] in the intervention arm vs 369 of 424 patients [87.0%] in the standard care arm), with no significant between-arm differences (aRR, 1.0; 95% CI, 0.9-1.1).

Patients would similarly recommend the communication approach used during the clinical encounter across clinical trial arms (390 of 429 encounters [90.9%] in the intervention arm and 378 of 425 encounters [88.9%] in the standard care arm). More clinicians were satisfied with the encounter in the intervention arm (400 of 453 encounters [88.3%]) compared with the standard care arm (277 of 448 encounters [61.8%]; aRR, 1.49; 95% CI, 1.42-1.53), and they were more likely to recommend using the SDM tool (396 of 453 encounters [87.4%]) than the standard care approach (199 of 448 encounters [44.4%]; aRR, 2.1; 95% CI, 2.0-2.2) to their colleagues. These results indicated good performance relative to the use of a per-protocol analyses or multiple imputation analyses for missing data rather than an intention-to-treat analysis (eTable 2 in Supplement 2).

### Observed Encounter Outcomes

Clinician involvement of patients in decision-making about anticoagulant treatment was significantly greater in the intervention arm compared with the standard care arm (OPTION12 mean [SD] score, 33.0 [10.8] points vs 29.1 [13.1] points, respectively; adjusted mean between-arm difference, 4.2 points; 95% CI, 2.8-5.6 points) (Table 4). Clinicians used the SDM tool with high fidelity (mean [SD] score, 5.6 [1.4] points of 7.0 possible points). However, the conversation focused first on the issue (eg, risk of bleeding, need for monitoring, or costs) identified by the patient as the highest priority in only 53 of 419 encounters [12.7%] in which the SDM tool was used. No significant difference was found in the duration of encounters between the intervention and standard care arms (mean [SD] duration, 32 [16] minutes vs 31 [17] minutes, respectively; adjusted mean between-arm difference, 1.1-minute; 95% CI, −0.3 to 2.5 minutes).

**Table 4.** Observed Encounter Outcomes

Outcome	No. (%)		Effect (95% CI)	Intracluster correlation	
	Intervention arm (n = 419)	Standard care arm (n = 411)		Clinic	Clinician/clinic
OPTION12 patient engagement score, mean (SD)	33.0 (10.8)	29.1 (13.1)	4.2 (2.8 to 5.6)	0.30	0.33
Fidelity score^a^					
Mean (SD)	5.6 (1.4)	0.2 (0.9)	NA	NA	NA
Median (IQR)	6.0 (6.0-6.0)	0	NA	NA	NA
Fidelity score components					
Tool was used^b^	401 (95.7)	9 (2.2)	NA	NA	NA
Tool sections used					
Current risk^c^	399 (95.2)	9 (2.2)	NA	NA	NA
Treated risk^d^	389 (92.8)	8 (1.9)	NA	NA	NA
Issues^e^	361 (86.2)	7 (1.7)	NA	NA	NA
Bleeding	367 (87.6)	320 (77.9)	NA	NA	NA
Anticoagulant treatment routine	373 (89.0)	306 (74.5)	NA	NA	NA
Reversing anticoagulant treatment	333 (79.5)	195 (47.4)	NA	NA	NA
Cost	378 (90.2)	261 (63.5)	NA	NA	NA
Diet and/or drug interaction	345 (82.3)	233 (56.7)	NA	NA	NA
How tool was used					
Presentation^f^	28 (6.7)	0	NA	NA	NA
Interaction^g^	359/401 (89.5)	9/9 (100)^h^	NA	NA	NA
Discussion was led by patient priority^i^	53 (12.7)	29 (7.1)	2.0 (1.3 to 3.2)^j^	0	0.44
Duration of encounter, mean (SD), min	32 (16)	31 (17)	1.1 (−0.3 to 2.5)^k^	0.08	0.63

Abbreviations: IQR, interquartile range; NA, not applicable; OPTION12, Observing Patient Involvement in Decision Making 12-item scale.

^a^ Fidelity score range, 0 to 7, with higher scores indicating greater fidelity.

^b^ Clear visual and/or contextual evidence indicated that the tool was used by the clinician (1 point possible).

^c^ A risk calculator was used to assess the patient’s current risk (1 point possible).

^d^ A risk calculator was used to assess the patient’s future risk after anticoagulant treatment (1 point possible).

^e^ Issue cards were presented to the patient (1 point possible).

^f^ The tool was presented to the patient without interaction (1 point possible).

^g^ The clinician interacted with the patient while using the tool to aid decision-making (2 points possible).

^h^ Contamination occurred owing to the use of the SDM tool in the standard care arm.

^i^ The discussion first addressed the issue of greatest salience (ie, the highest priority) to the patient.

^j^ Relative risk. Adjusted by treatment arm, with the random effect of clinic and clinician.

^k^ Mean difference between the intervention and standard care arms.

Overall, a median of 2 patients (interquartile range [IQR], 1-6 patients; range, 1-76 patients) were enrolled per clinician, with a median of 1 patient (range, 1-38 patients) per clinician in the standard care arm and 2 patients (range, 1-44 patients) per clinician in the intervention arm. Of 151 clinicians who had encounters with 1 or more patients enrolled in the study, 68 clinicians enrolled patients in both arms of the clinical trial (median, 7 patients per clinician; IQR, 3-14 patients per clinician). Minimal data were found to indicate clinicians’ use of the SDM tool in the standard care arm or contamination of SDM behaviors between arms (data not shown).

## Discussion

### Main Findings

This encounter-level multicenter randomized clinical trial found that adding an SDM tool to standard care during clinical encounters with patients with AF improved several aspects of SDM quality without significantly affecting anticoagulant treatment decisions or lengthening the duration of the encounters. Clinicians who used the SDM tool were significantly more likely to engage patients in SDM and to be more satisfied with the encounters in which they used the SDM tool.

To date, 3 clinical trials have tested the effect of using SDM tools to facilitate real-life decisions about anticoagulant treatment in patients with AF; those clinical trials yielded inconsistent results with regard to patient knowledge, decisional conflict, and anticoagulant treatment choices.^9^ Compared with those studies, the present clinical trial combined the evaluation of an SDM tool that supported both patients and clinicians in deciding how to prevent strokes (including the option to receive DOAC medications), rather than supporting patients alone, with an assessment of the intervention’s effectiveness through the use of recorded encounters.

Results from the present clinical trial are consistent with previous SDM clinical trials conducted by the Knowledge and Evaluation Research Unit at Mayo Clinic,^32,33,34^ with the exception of the lack of significant improvements in patient knowledge or decisional conflict (which was nearly optimal at baseline). Compared with the findings of 105 randomized clinical trials of SDM interventions that were included in a 2017 Cochrane systematic review,^10^ the present clinical trial yielded similar results, with the exception of no significant improvements in patient knowledge or decisional conflict (both were reported to improve in the review) and no significant change in encounter duration (reported to lengthen by 3 minutes in the review). Overall, the present study is, to our knowledge, one of the largest SDM clinical trials conducted and one of the few to intervene in the clinical encounter and directly observe SDM behaviors, fidelity of use, and contamination.

### Implications

This clinical trial demonstrates a feasible and acceptable approach to implementing an SDM tool to guide anticoagulant treatment discussions during clinical encounters with patients with AF in diverse practice settings, and it provides credible estimates of the efficacy of the SDM approach. This approach also offers a way to bring risk assessment (in this case, through use of the CHA_2_DS_2_-VASc score) into the patient-centered decision-making process.^35^ During the clinical trial, the SDM recommendation for encounters with patients with AF shifted from discussing warfarin and DOAC medications as treatment choices to discussing only DOAC medications as treatment choices.^36^ This change may have reduced the scope of SDM for decisions about how to treat patients for anticoagulation and may have limited SDM application to the decision of whether to treat patients for anticoagulation. Further research may be necessary to understand the extent to which current anticoagulant treatment decisions are inappropriate across clinical and patient factors.

Whether a more selective implementation approach could yield larger effects remains unclear and deserves examination. Such an examination may need to focus on patients who may find it difficult to decide whether or how to use anticoagulant treatments, such as patients with low to intermediate stroke risk, patients who have experienced difficulty maintaining therapeutic international normalized ratio levels, or patients who find DOAC medications unaffordable. Some explorations are currently occurring in an ongoing multicenter randomized clinical trial funded by the American Heart Association and the Patient Centered Outcomes Research Institute that compares standard care, an SDM encounter tool, a patient decision aid, or a combination of these options.^37^

The findings of this clinical trial contribute to the discussion of the recommendations for SDM in published guidelines, which inspired this clinical trial, and the mandated use of SDM by payers as a requisite for reimbursement. Both practices assume that clinicians and health care systems can implement forms of SDM that are capable of responding effectively to the problematic situation for which patients seek care.^38^ The finding that the patient’s highest priority led the discussion in only a limited number of SDM encounters challenges this assumption. There is a clinical and ethical need for patients and clinicians to work together to form plans of care. How best to do so remains to be determined. Furthermore, it remains necessary to develop ways of identifying which patients, decisions, encounters, and clinicians need more support to enact which form of SDM.^39^ The results of the present study suggest it may be premature to proceed with a wholesale implementation of SDM tools that is sustained by mandates or financial incentives.

### Strengths and Limitations

This study had several strengths. Some aspects of the conduct of the clinical trial contribute to the credibility of its findings. The implementation of allocation concealment and adherence to the intention-to-treat principle in the conduct and analyses of the study mitigated the intrusion of bias. Video review demonstrated that, in most cases, the clinicians used the intervention correctly, and no substantial contamination occurred. This clinical trial, one of the largest clinical trials of SDM to date,^10^ has yielded precise estimates of effect.

The study also had several limitations. Some features of the clinical trial may have contributed to an overestimation of the effect of the SDM intervention. Selection bias could have been introduced when enrolled clinicians chose not to enroll an eligible patient encounter into the clinical trial. In addition, bias may have affected the unblinded assessment of recorded encounters and the scoring of those encounters using the OPTION12 scale. Some features of the clinical trial may alternately have produced underestimations of the effect of the SDM intervention. Based on the results found in the standard care arm, participants in the present study were particularly competent at implementing SDM compared with those in previous studies.^40^ In addition, patients who were already receiving anticoagulant treatment (ie, those enrolled in the review cohort), who comprised most of the clinical trial participants, may have been generally satisfied with their current anticoagulant treatment regimen or may have had no difficulty deciding on their best course of action (ie, they already had high levels of knowledge and low levels of decisional conflict at baseline). Both factors may have limited the potential contribution of the SDM tool, although no significant treatment-outcome interactions by clinic or cohort (start vs review) were found.

## Conclusions

The use of an encounter tool to foster and support SDM resulted in improvements in several aspects of SDM quality and clinician satisfaction, with no significant effect on treatment decisions or encounter duration. These results question the view that implementing SDM tools for anticoagulant treatment can improve care for patients with AF.

## Notes

Supplement 1. Trial Protocol

Supplement 2. **eMethods.** Clinician Preencounter and Postencounter Survey and Patient Postencounter Survey **eTable 1.** Interaction of Treatment and Prespecified Factors for SDM Outcomes **eTable 2.** Quality of Shared Decision-Making: Sensitivity Analysis

Supplement 3. Data Sharing Statement
