# Supplementary material for: Assessment of Shared Decision-making for Stroke Prevention in Patients With Atrial Fibrillation: A Randomized Clinical Trial
Source: JAMA Intern Med. 2020 Jul 20;180(9):1–10. doi: 10.1001/jamainternmed.2020.2908 (PMC7372497; doi:10.1001/jamainternmed.2020.2908)
Supplement: Supplement 2. — eMethods. Clinician Preencounter and Postencounter Survey and Patient Postencounter Survey eTable 1. Interaction of Treatment and Prespecified Factors for SDM Outcomes eTable 2. Quality of Shared Decision-Making: Sensitivity Analysis [file jamainternmed-e202908-s002.pdf]

Supplementary Online Content

Kunneman M, Branda ME, Hargraves IG, et al; Shared Decision Making for Atrial Fibrillation (SDM4AFib) Trial Investigators. Assessment of shared decision-making for stroke prevention in patients with atrial fibrillation: a randomized clinical trial. *JAMA Intern Med*. Published July 20, 2020. doi:10.1001/jamainternmed.2020.2908

eMethods. Clinician Preencounter and Postencounter Survey and Patient Postencounter Survey

eTable 1. Interaction of Treatment and Prespecified Factors for SDM Outcomes

eTable 2. Quality of Shared Decision-Making: Sensitivity Analysis

This supplementary material has been provided by the authors to give readers additional information about their work.

© 2020 Kunneman M et al. *JAMA Internal Medicine*.

eMethods

Clinician pre- and post-encounter survey and patient post-encounter survey

© 2020 Kunneman M et al. *JAMA Internal Medicine*.

Clinician Study ID: ____________________

1. **Today's Date:** __ __/__ __/__ __ __ __
   Month Day Year

2. **Are you:** 1☐ Male 2☐ Female

3. **In what year were you born?** 19 __ __

4. **What is your degree?**
   - 1☐ Nurse practitioner
   - 2☐ Physician assistant
   - 3☐ Medical doctor
   - 4☐ Osteopathic doctor
   - 5☐ Pharmacist
   - 6☐ Other, please specify: ____________________

5. **Please indicate which of the following is your practice:**
   - 1☐ Family Medicine
   - 2☐ Internal Medicine
   - 3☐ Cardiology
   - 4☐ Cardiac Electrophysiology
   - 5☐ Pharmacy
   - 6☐ Other, please specify: ____________________

6. **Are you a resident/fellow?**
   1☐ Yes 2☐ No

   If no, how many total years have you been in practice after completing all training (residency and fellowship)?

   ___ ___ Years

7. **How many years have you worked in this practice setting?**
   - 1☐ Less than one year
   - 2☐ 1-4 years
   - 3☐ 5-8 years
   - 4☐ More than 8 years

© 2020 Kunneman M et al. *JAMA Internal Medicine*.

8. **In your estimation, how many patients do you evaluate per week who are considering or currently taking an anticoagulant? Please provide a value rather than a range.**

   __ __ __ **Patients**

9. **I feel burned out from work.**
   - 1 ☐ Never
   - 2 ☐ A few times a year or less
   - 3 ☐ Once a month or less
   - 4 ☐ A few times a month
   - 5 ☐ Once a week
   - 6 ☐ A few times a week
   - 7 ☐ Every day

10. **I've become more callous toward people since I took this job.**
   - 1 ☐ Never
   - 2 ☐ A few times a year or less
   - 3 ☐ Once a month or less
   - 4 ☐ A few times a month
   - 5 ☐ Once a week
   - 6 ☐ A few times a week
   - 7 ☐ Every day

The following questions are designed to help get a better understanding of how to apply and integrate shared decision-making interventions in health care. Please take the time to decide which answer best suits your experience for each statement and tick the appropriate response. This survey asks questions about anticoagulation choice decision aid.

11. **When you use anticoagulation choice, <u>how familiar</u> does it feel?** (Please mark one.)

   0 ☐ 1 ☐ 2 ☐ 3 ☐ 4 ☐ 5 ☐ 6 ☐ 7 ☐ 8 ☐ 9 ☐ 10 ☐

   Still feels very new — Feels completely familiar

12. **Do you feel anticoagulation choice, <u>is currently</u> a normal part of your work?** (Please mark one.)

   0 ☐ 1 ☐ 2 ☐ 3 ☐ 4 ☐ 5 ☐ 6 ☐ 7 ☐ 8 ☐ 9 ☐ 10 ☐

   No, not at all — Yes, very much so

13. **Do you feel anticoagulation choice, <u>will become</u> a normal part of your work?** (Please mark one.)

   0 ☐ 1 ☐ 2 ☐ 3 ☐ 4 ☐ 5 ☐ 6 ☐ 7 ☐ 8 ☐ 9 ☐ 10 ☐

   No, not at all — Yes, very much so

© 2020 Kunneman M et al. *JAMA Internal Medicine*.

14. **For each statement below, please select an answer that best suits your experience using Option A. If the statement is not relevant to you, please select an answer from Option B.**

	Option A: Strongly agree	Option A: Agree	Option A: Neither agree or disagree	Option A: Disagree	Option A: Strongly disagree	Option B: Not relevant to my role	Option B: Not relevant at this stage	Option B: Not relevant to the intervention
**a. I can see how anticoagulation choice differs from usual ways of working.**	1 ☐	2 ☐	3 ☐	4 ☐	5 ☐	1 ☐	2 ☐	3 ☐
**b. Staff in this organization have a shared understanding of the purpose of anticoagulation choice.**	1 ☐	2 ☐	3 ☐	4 ☐	5 ☐	1 ☐	2 ☐	3 ☐
**c. I understand how anticoagulation choice affects the nature of my own work.**	1 ☐	2 ☐	3 ☐	4 ☐	5 ☐	1 ☐	2 ☐	3 ☐
**d. I can see the potential value of anticoagulation choice for my work.**	1 ☐	2 ☐	3 ☐	4 ☐	5 ☐	1 ☐	2 ☐	3 ☐
**e. There are key people who drive anticoagulation choice forward and get others involved.**	1 ☐	2 ☐	3 ☐	4 ☐	5 ☐	1 ☐	2 ☐	3 ☐
**f. I believe that participating in anticoagulation choice is a legitimate part of my role.**	1 ☐	2 ☐	3 ☐	4 ☐	5 ☐	1 ☐	2 ☐	3 ☐
**g. I'm open to working with colleagues in new ways to use anticoagulation choice.**	1 ☐	2 ☐	3 ☐	4 ☐	5 ☐	1 ☐	2 ☐	3 ☐
**h. I will continue to support anticoagulation choice.**	1 ☐	2 ☐	3 ☐	4 ☐	5 ☐	1 ☐	2 ☐	3 ☐
**i. I can easily integrate anticoagulation choice into my existing work.**	1 ☐	2 ☐	3 ☐	4 ☐	5 ☐	1 ☐	2 ☐	3 ☐
**j. Anticoagulation choice disrupts working relationships.**	1 ☐	2 ☐	3 ☐	4 ☐	5 ☐	1 ☐	2 ☐	3 ☐
**k. I have confidence in other people's ability to use anticoagulation choice.**	1 ☐	2 ☐	3 ☐	4 ☐	5 ☐	1 ☐	2 ☐	3 ☐

Continued next page...

© 2020 Kunneman M et al. *JAMA Internal Medicine*.

	Option A: Strongly agree	Option A: Agree	Option A: Neither agree or disagree	Option A: Disagree	Option A: Strongly disagree	Option B: Not relevant to my role	Option B: Not relevant at this stage	Option B: Not relevant to the intervention
**l. Work is assigned to those with skills appropriate to anticoagulation choice.**	1 ☐	2 ☐	3 ☐	4 ☐	5 ☐	1 ☐	2 ☐	3 ☐
**m. Sufficient training is provided to enable staff to implement anticoagulation choice..**	1 ☐	2 ☐	3 ☐	4 ☐	5 ☐	1 ☐	2 ☐	3 ☐
**n. Sufficient resources are available to support anti-coagulation choice.**	1 ☐	2 ☐	3 ☐	4 ☐	5 ☐	1 ☐	2 ☐	3 ☐
**o. Management adequately supports anticoagulation choice..**	1 ☐	2 ☐	3 ☐	4 ☐	5 ☐	1 ☐	2 ☐	3 ☐
**p. I am aware of reports about the effects of anti-coagulation choice..**	1 ☐	2 ☐	3 ☐	4 ☐	5 ☐	1 ☐	2 ☐	3 ☐
**q. The staff agree that anti-coagulation choice is worthwhile.**	1 ☐	2 ☐	3 ☐	4 ☐	5 ☐	1 ☐	2 ☐	3 ☐
**r. I value the effects that anti-coagulation choice has had on my work.**	1 ☐	2 ☐	3 ☐	4 ☐	5 ☐	1 ☐	2 ☐	3 ☐
**s. Feedback about anticoagu-lation choice can be used to improve it in the future.**	1 ☐	2 ☐	3 ☐	4 ☐	5 ☐	1 ☐	2 ☐	3 ☐
**t. I can modify how I work with anticoagulation choice.**	1 ☐	2 ☐	3 ☐	4 ☐	5 ☐	1 ☐	2 ☐	3 ☐

Thank you for your time.

Please return the completed survey to the study coordinator.

MC4269-842rev0417

© 2020 Kunneman M et al. *JAMA Internal Medicine*.

____________________ **Clinician Study ID**

____________________ **Patient Study ID**

Today's Date: __ __/__ __/__ __ __ __
Month Day Year

Shared Decision-Making for Stroke Prevention in Atrial Fibrillation (SDM4Afib)

Thank you for taking the time to complete this survey. Your answers are very important to us. <u>Thinking about the conversation you just had with your patient about anticoagulation medications,</u> please read and answer each question. Your responses are confidential.

1. What decision did you and your patient make today regarding anticoagulation medication?

1 ☐ To start or continue on Warfarin
2 ☐ To start or continue on apixaban (Eliquis), dabigatran (Pradaxa), edoxaban (Savaysa), or rivaroxaban (Xarelto)
3 ☐ To not take an anticoagulation medication
4 ☐ To start taking aspirin or other antiplatelet agent for the purpose of stroke prevention
5 ☐ To make the decision at some other time
6 ☐ No discussion about anticoagulation medication took place
7 ☐ Other, please specify: ____

2. To what extent do you think the decision about anticoagulation medication was already made prior to this visit?

1 ☐	2 ☐	3 ☐	4 ☐	5 ☐	6 ☐	7 ☐
Not at all						Very much

3. Who do you feel had the most say in deciding about anticoagulation medication?

1 ☐ The patient (and his/her spouse/family member/friend)
2 ☐ The referring clinician
3 ☐ Myself (and my team)

4. To what extent did you feel satisfied with the discussions you just had with your patient about taking an anticoagulant?

1 ☐	2 ☐	3 ☐	4 ☐	5 ☐	6 ☐
NA/no discussion	Not at all satisfied		Somewhat satisfied		Completely satisfied

5. Would you recommend to other clinicians the way you and your patient worked together today to make a decision about taking an anticoagulant?

1 ☐	2 ☐	3 ☐	4 ☐	5 ☐	6 ☐	7 ☐	8 ☐
NA/ No discussion	No, I would strongly recommend against it			Not sure whether to recommend it or not			Yes, I would strongly recommend it

© 2020 Kunneman M et al. *JAMA Internal Medicine*.

MAYO
CLINIC

Shared Decision-Making for Stroke Prevention in Atrial Fibrillation

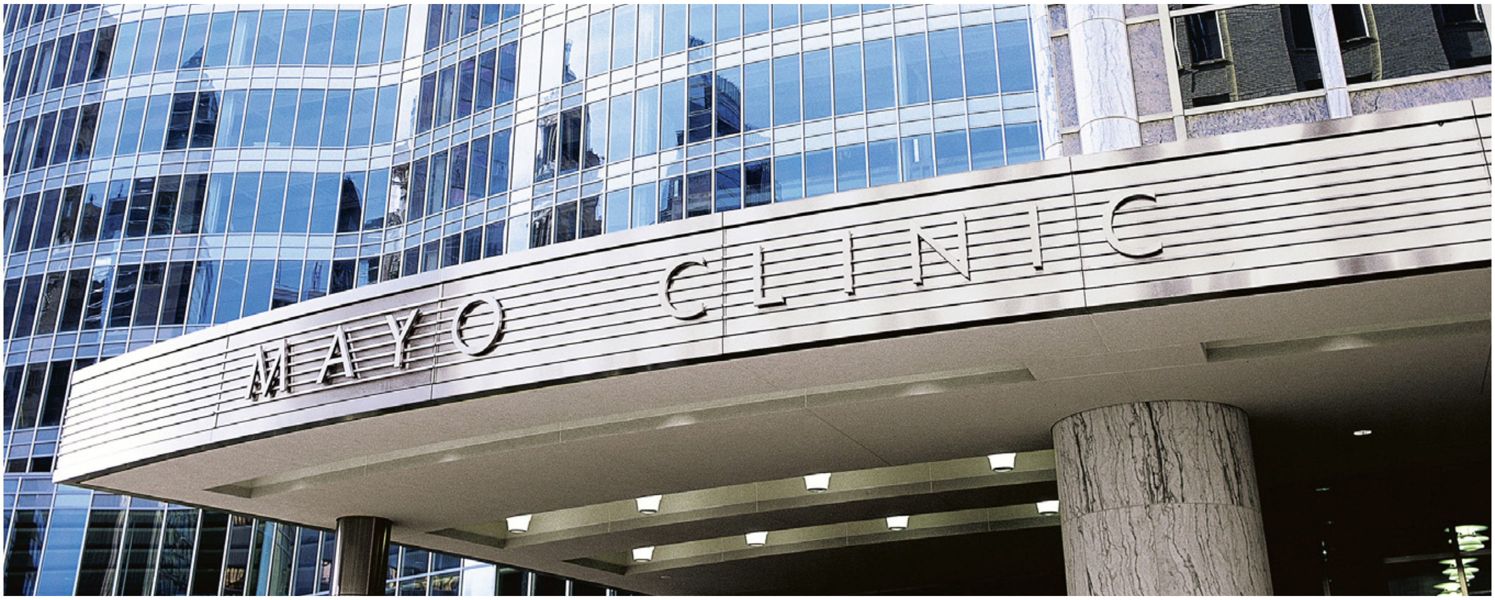

Survey Research Center

© 2020 Kunneman M et al. *JAMA Internal Medicine*.

Thank you for taking the time to complete this survey. Your answers are important to us. Please take the time to read and answer each question carefully by marking the box or providing the response that best represents your answers. Your responses are confidential and your clinician will not see your answers.

After completing this survey, please return it to the clinical staff that gave it to you. Please feel free to ask any questions you have while completing the survey.

Thank you.

© 2020 Kunneman M et al. *JAMA Internal Medicine*.

Patient ID: __ __ __ __ __ __ __

INSTRUCTIONS: PLEASE CHECK THE APPROPRIATE BOX OR FILL IN THE BLANK AS INDICATED.

1. **Today's Date:** __ __/__ __/__ __ __ __
   Month Day Year

Below are some statements about blood thinners (anticoagulation medicines). Please show whether you think they are correct (True), not correct (False), or you are unsure (Do not know). This is not a test - what is important is that your answers reflect what you think is true about blood thinners (anticoagulation medicines).

	True	False	Do not know
2. **Taking blood thinners can lower my risk of stroke.**	1 ☐	2 ☐	3 ☐
3. **Taking blood thinners can increase my risk of bleeding.**	1 ☐	2 ☐	3 ☐
4. **Some, but not all, blood thinners require me to get periodic blood tests.**	1 ☐	2 ☐	3 ☐
5. **Blood thinners can be used to treat a stroke if I have one.**	1 ☐	2 ☐	3 ☐
6. **Blood thinners must be taken daily.**	1 ☐	2 ☐	3 ☐
7. **I can safely eat whatever I want when taking Warfarin.**	1 ☐	2 ☐	3 ☐

8. **Of 100 people like me, the number expected to have a disabling or fatal stroke in the next year is: Provide a value between 0 - 100.**

   __ __ __ 1 ☐ I don't know

9. **Which decision did you and your clinician make today about taking a blood thinner?** (Please mark one.)

   1 ☐ Start or continue taking the blood thinner Warfarin
   2 ☐ Start or continue taking one of the blood thinners known as direct anticoagulants: apixaban (Eliquis), dabigatran (Pradaxa), edoxaban (Savaysa), or rivaroxaban (Xarelto)
   3 ☐ Stop taking or not start any blood thinner
   4 ☐ Make a decision at some other time
   5 ☐ Other decision, please specify: ______________________________

10. **Was the cost of the blood thinner (anticoagulant medicine) a factor in your decision?**

   1 ☐ No, cost did not matter
   2 ☐ Yes, cost was one factor I considered in my decision
   3 ☐ Yes, cost was the sole factor in my decision

© 2020 Kunneman M et al. *JAMA Internal Medicine*.

Now, thinking about the decision you made with your clinician about blood thinners today, please look at the following comments that some people have made when deciding about blood thinners to lower their risk of stroke. Please show how strongly you agree or disagree with these comments by checking the box that indicates your level of agreement.

	Strongly agree	Agree	Neither agree nor disagree	Disagree	Strongly disagree
**11. I know which options are available to me.**	1 ☐	2 ☐	3 ☐	4 ☐	5 ☐
**12. I know the benefits of each option.**	1 ☐	2 ☐	3 ☐	4 ☐	5 ☐
**13. I know the risks and side effects of each option.**	1 ☐	2 ☐	3 ☐	4 ☐	5 ☐
**14. I am clear about which benefits matter most to me.**	1 ☐	2 ☐	3 ☐	4 ☐	5 ☐
**15. I am clear about which risks and side effects matter most to me.**	1 ☐	2 ☐	3 ☐	4 ☐	5 ☐
**16. I am clear about which is more important to me (the benefits or the risks and side effects).**	1 ☐	2 ☐	3 ☐	4 ☐	5 ☐
**17. I have enough support from others to make a choice.**	1 ☐	2 ☐	3 ☐	4 ☐	5 ☐
**18. I am choosing without pressure from others.**	1 ☐	2 ☐	3 ☐	4 ☐	5 ☐
**19. I have enough advice to make a choice.**	1 ☐	2 ☐	3 ☐	4 ☐	5 ☐
**20. I am clear about the best choice for me.**	1 ☐	2 ☐	3 ☐	4 ☐	5 ☐
**21. I feel sure about what to choose.**	1 ☐	2 ☐	3 ☐	4 ☐	5 ☐
**22. This decision is easy for me to make.**	1 ☐	2 ☐	3 ☐	4 ☐	5 ☐
**23. I feel I have made an informed choice.**	1 ☐	2 ☐	3 ☐	4 ☐	5 ☐
**24. I expect to stick with my decision.**	1 ☐	2 ☐	3 ☐	4 ☐	5 ☐
**25. The decision shows what is important to me.**	1 ☐	2 ☐	3 ☐	4 ☐	5 ☐
**26. I am satisfied with my decision.**	1 ☐	2 ☐	3 ☐	4 ☐	5 ☐

© 2020 Kunneman M et al. *JAMA Internal Medicine*.

Thinking of the conversation you just had with your clinician about blood thinners (anticoagulation medicines), please select the most appropriate response to each item below:

	Yes, definitely	Yes, somewhat	No
**27. Did your clinician explain things in a way that was easy to understand?**	1 ☐	2 ☐	3 ☐
**28. Did your clinician listen carefully to you?**	1 ☐	2 ☐	3 ☐
**29. Did your clinician show respect for what you had to say?**	1 ☐	2 ☐	3 ☐

30. Would you recommend the way that you and your clinician shared information about your blood thinners (anticoagulation medicines) to other patients?

1 ☐	2 ☐	3 ☐	4 ☐	5 ☐	6 ☐	7 ☐
Yes, I would strongly recommend it			Not sure whether to recommend it or not			No, I would strongly recommend against it

31. In general, would you say your health is:

1 ☐ Excellent 2 ☐ Very good 3 ☐ Good 4 ☐ Fair 5 ☐ Poor

32. How many different medicines in total, prescription and over-the-counter, do you take per day? (Please count a medicine taken several times per day as one medicine.)

__ __ Number of different medicines per day 1 ☐ I do not take any medicines

33. Are you currently taking any of the following medications?

1 ☐ Aspirin
2 ☐ NSAIDS (ibuprofen/Advil, naproxen/Aleve, indomethacin/Indocin, diclofenac/Voltaren)
3 ☐ Antiplatelet agents (clopidogrel/Plavix, ticagrelor/Brilinta, prasugrel/Effient)
4 ☐ No, I do not take any of these medications currently

 © 2020 Kunneman M et al. *JAMA Internal Medicine*.

34. Do you have a medical condition that increases your chance of bleeding?
(i.e., anemia, history of gastric or intestinal ulcers, diverticulosis, hemophelia)

1 ☐ No 2 ☐ Yes

If yes, which of the bleeding conditions do you have?
(Mark all that apply.)

1 ☐ Anemia
1 ☐ History of gastric or intestinal ulcers
1 ☐ Diverticulosis
1 ☐ Inherited condition of the blood system such as hemophilia
1 ☐ Other, please specify: ________________________________

35. In a typical week (7-day period), how many alcoholic drinks do you consume?
(One drink is 12 ounces of beer, 5 ounces of wine or 1.5 ounces of hard alcohol.)

__ __ Drinks per week

36. Have you ever had a bleeding event that required hospitalization?

1 ☐ No 2 ☐ Yes

If yes,

When was the most recent event: __ __/__ __ __ __
Month Year

What was the nature of the event: ________________________________

37. Have you ever suffered a fall that has resulted in a major bleed, broken bone, head injury, or required hospitalization?

1 ☐ No 2 ☐ Yes

If yes, what was the nature of the most recent fall?

____

38. Do you reside in a long-term care facility (i.e., nursing home)?

1 ☐ No 2 ☐ Yes

39. Do you receive your primary health care needs at another healthcare organization than this one?

1 ☐ No 2 ☐ Yes

© 2020 Kunneman M et al. *JAMA Internal Medicine*.

FOR EACH OF THE FOLLOWING QUESTIONS, PLEASE CHECK THE BOX THAT BEST REFLECTS YOUR ANSWER.

40. How confident are you filling out medical forms by yourself?

1 ☐ Not at all 2 ☐ A little bit 3 ☐ Somewhat 4 ☐ Quite a bit 5 ☐ Extremely

41. When reading the newspaper, how helpful do you find tables and graphs that are parts of a story?

1 ☐	2 ☐	3 ☐	4 ☐	5 ☐	6 ☐
Not at all helpful					Extremely helpful

42. When people tell you the chance of something happening, do you prefer that they use words ("it rarely happens") or numbers ("there's a 1% chance")?

1 ☐	2 ☐	3 ☐	4 ☐	5 ☐	6 ☐
Always prefer words					Always prefer numbers

43. When you hear a weather forecast, do you prefer predictions using percentages (e.g., "there will be a 20% chance of rain today") or predictions using only words (e.g., "there is a small chance of rain today")?

1 ☐	2 ☐	3 ☐	4 ☐	5 ☐	6 ☐
Always prefer percentages					Always prefer words

44. How often do you find numerical information to be useful?

1 ☐	2 ☐	3 ☐	4 ☐	5 ☐	6 ☐
Never					Very often

© 2020 Kunneman M et al. *JAMA Internal Medicine*.

Some questions about you . . .

45. Are you currently: (Please mark one.)

1 ☐ Married
2 ☐ Living with someone in a marriage-like relationship
3 ☐ Separated
4 ☐ Divorced
5 ☐ Widowed
6 ☐ Never married

46. What is the highest level of schooling you have completed? (Please mark one.)

1 ☐ Some high school or less
2 ☐ High school graduate or GED
3 ☐ Some college or associate's degree (including community college) or vocational technical, or business school degree
4 ☐ Four-year college graduate (bachelor's degree)
5 ☐ Graduate or professional school degree
6 ☐ Other, please specify: ______________________________

47. Which of the following categories best describes your household income last year before taxes?

1 ☐ Less than $20,000
2 ☐ $20,000 to $29,999
3 ☐ $30,000 to $39,999
4 ☐ $40,000 to $59,999
5 ☐ $60,000 to $79,999
6 ☐ $80,000 to $99,999
7 ☐ $100,000 to $149,999
8 ☐ $150,000 to $199,999
9 ☐ $200,000 or more

48. Are you of Hispanic, Latino, or Spanish origin?

1 ☐ Yes (Mexican, Mexican American, Chicano, Puerto Rican, Cuban, Other)
2 ☐ No, not Hispanic, Latino, or Spanish origin

49. What is your race? (Mark all that apply.)

1 ☐ White
1 ☐ Black or African American
1 ☐ American Indian or Alaska Native
1 ☐ Asian
1 ☐ Native Hawaiian or other Pacific Islander
1 ☐ Other, please specify: ______________________________

© 2020 Kunneman M et al. *JAMA Internal Medicine*.

Thank you for completing the survey!

Please return it to the clinical support staff that asked you to complete it.

Questions 11-26: AM O'Connor, Decisional Conflict Scale. Copyright © 1993 [updated 2005]. Available from www.ohri.ca/decisionaid

© 2020 Kunneman M et al. *JAMA Internal Medicine*.

eTable 1. Interaction of treatment and pre-specified factors for SDM Outcomes

Outcome	Interaction Term	Chi-Square Statistic	Degrees of freedom	*p*	Corrected *p*-value[1]
**Knowledge transfer**					
Knowledge (Correct answers, out of 6)	CHA_2DS_2-VASc	1	1	0.32	0.63
	Numeracy	0.04	1	0.84	>0.99
	Academic	0.3	3	0.96	0.96
	Cohort	1	1	0.32	>0.99
Risk estimation					
Strict Score (±10%)	CHA_2DS_2-VASc	0.14	1	0.71	0.98
	Numeracy	0.36	1	0.55	>0.99
	Academic	3.2	3	0.36	>0.99
	Cohort	1.04	1	0.31	>0.99
Liberal Score (±30%)	CHA_2DS_2-VASc	0.6	1	0.44	0.79
	Numeracy	4.4	1	0.04	0.28
	Academic	1.54	3	0.67	>0.99
	Cohort	2.56	1	0.11	>0.99
**Decision concordance**					
	CHA_2DS_2-VASc	2.98	1	0.08	0.17
	Numeracy	0.52	1	0.47	0.63
	Academic	0	3	>0.99	>0.99
	Cohort	3.94	1	0.05	0.19
**Decision satisfaction**					

© 2020 Kunneman M et al. *JAMA Internal Medicine*.

Decisional Conflict Scale Overall Score	CHA_2DS_2-VASc	1.78	1	0.18	0.73
	Numeracy	1.6	1	0.21	0.41
	Academic	1.8	3	0.61	0.82
	Cohort	0.04	1	0.84	0.84
Patient recommend way information shared	CHA_2DS_2-VASc	0.4	1	0.53	0.70
	Numeracy	6.08	1	0.01	0.05
	Academic	6.24	3	0.10	0.20
	Cohort	0.06	1	0.81	0.81
Clinician recommend way information shared	CHA_2DS_2-VASc	2.7	1	0.10	0.20
	Numeracy	0.66	1	0.42	0.56
	Academic	1.46	3	0.69	0.69
	Cohort	5.34	1	0.02	0.08
Clinician Satisfied with Conversation	CHA_2DS_2-VASc	3.6	1	0.06	0.23
	Numeracy	0.54	1	0.46	0.62
	Academic	0.42	3	0.94	0.94
	Cohort	2.58	1	0.11	0.22
**Clinician involvement of patients in SDM**					
OPTION 12	CHA_2DS_2-VASc	1.26	1	0.26	0.26
	Numeracy	2.08	1	0.15	0.60
	Academic	5.06	3	0.17	0.33
	Cohort	1.42	1	0.23	0.31

Cutoffs used: CHA_2DS_2-VASc score: 1 or ≥ 2 for men vs. 2 or ≥ 3 for women; Numeracy (Subjective Numeracy Scale): Score less than 4 vs. ≥ 4; Academic: Academic Center vs. Community vs. Safety Net; Cohort: Start vs. Review.

1 - Benjamini-Hochberg method adjusted *p* value

© 2020 Kunneman M et al. *JAMA Internal Medicine*.

eTable 2. Quality of shared decision making. Sensitivity Analysis

	Per Protocol Analysis[1]	Multiple Imputation
**Outcome**	**Mean difference (95% CI)**	**Mean difference (95% CI)**
**Knowledge transfer**		
Knowledge (correct answers, out of 6)[2]	RR 1.01 (0.99, 1.02)	1.01 (0.99, 1.02)
Risk estimation		
Strict Score (±10%)[2]	RR 1.4 (0.8, 2.3)	1.3 (0.8, 2.1)
Liberal Score (±30%)[2]	RR 1.3 (0.82, 1.83)	1.2 (0.8, 1.7)
**Decision satisfaction**		
Decisional Conflict Scale, overall score	-.1 (-3.1, 0.8)	-1.2 (-3.1, 0.7)
Patient recommend way information shared[2]	RR1.02 (0.96, 1.06)	1.02 (0.97, 1.06)
Clinician recommend way information shared[2]	RR 2.1 (2.04, 2.18)	2.1 (2.03, 2.17)
Clinician satisfied with conversation[2]	RR 1.49 (1.42, 1.53)	1.48 (1.42, 1.52)

1 – All encounters without recording are included as determination of adherence to protocol cannot be determined, encounters with recording where tool was not used in the intervention arm (N=13) or the tool was used in the usual care arm (N=9) were removed from the analysis. Only complete cases were included.
2 – Relative Risk (RR) with 95% confidence interval, adjusted by arm, cohort (start vs. review), stroke risk (CHA_2DS_2-VASc 1 vs ≥2 for men and 1-2 vs. ≥3 for female) with random effect of clinic and clinician.

© 2020 Kunneman M et al. *JAMA Internal Medicine*.
