# Supplementary material for: Assessment of Shared Decision-making for Stroke Prevention in Patients With Atrial Fibrillation: A Randomized Clinical Trial
Source: JAMA Intern Med. 2020 Jul 20;180(9):1–10. doi: 10.1001/jamainternmed.2020.2908 (PMC7372497; doi:10.1001/jamainternmed.2020.2908)
Supplement: Supplement 3. — Data Sharing Statement [file jamainternmed-e202908-s003.pdf]

Data Sharing Statement

Kunneman. Assessment of Shared Decision-making for Stroke Prevention in Patients With Atrial Fibrillation. . Published July 20, 2020.

Data
Data available: Yes
Data types: Deidentified participant data, Participant data with identifiers
How to access data: montori.victor@mayo.edu - we are working with the NHLBI to make deidentified participant data available upon completion of AIM 2 of our grant (July 2020). Video of clinical encounters (a special case of data with identifiers) enter a video repository that includes all the videos that we have completed in RCTs over the last 15 years. These are also available (for review on site) under IRB approval.
When available: beginning date: 08-01-2020

Supporting Documents
Document types: Other (please specify)
Additional Information: In this multicenter trial, we have developed a writing group and this group reviews and prioritizes requests for secondary data analyses. Analytic code and informed consent forms are made available to anyone within the multicenter trial group first until 2022, but we will consider requests from outside after that time.
How to access documents: montori.victor@mayo.edu
When available: beginning date: 01-01-2023

Additional Information
Who can access the data: Anyone making a written request to the PI: montori.victor@mayo.edu
Types of analyses: Research proposals will be submitted to the PI and the writing team will review and prioritize these requests; these proposals can be for any purpose.
Mechanisms of data availability: Upon agreement to share data and with funding to the statistical team if needed to complete the project. There will be no requirement to fund the PI or any of the investigators otherwise.
